# Effect of Interventions on Iron-Deficiency Anemia Among School-Going Children in India: A Systematic Review and Network Meta-Analysis

**DOI:** 10.34172/jrhs.8985

**Published:** 2025-06-10

**Authors:** Flemin Felix, Kalesh Mappilakudy Karun, Chandan Nagendraswamy, Deepthy Melepurakkal Sadanandan, Yadu Damodaran, Manish Barvaliya, Subarna Roy

**Affiliations:** ^1^Department of Health Systems Research, ICMR – National Institute of Traditional Medicine, Nehru Nagar, Belagavi, 590010, Karnataka, India; ^2^Women’s and Children’s Health Research Unit, Jawaharlal Nehru Medical College, KLE Academy of Higher Education and Research, Belagavi, 590010, Karnataka, India; ^3^Department of Biostatistics, St. Thomas College, Palai, 686574, Kerala, India; ^4^Department of Health Research (Government of India), Model Rural Health Research Unit, Sirwar, Raichur, Karnataka, India

**Keywords:** Anemia, Haemoglobin, India, Network meta-analysis, Systematic review, School-going children

## Abstract

**Background:** The prevalence of iron-deficiency anemia (IDA) among school-aged children in India varies from 27% to 90%. There is no evidence of the comparative effects of various available interventions. Thus, this study aimed to quantify and rank the effects of different interventions on IDA among school-going children.

**Study Design:** Systematic review and Meta-analysis.

**Methods:** To this end, PubMed, Scopus, and ScienceDirect databases were searched, and randomized controlled trials (RCTs) evaluating the comparative effects of various interventions on hemoglobin (Hb) and serum ferritin against a control were included in this study. The random-effect model was conducted for Hb, and the fixed-effects model was performed for ferritin to estimate the mean difference (MD) and 95% confidence interval (CI) of the effect of interventions of outcomes based on the heterogeneity (I^2^).

**Results:** Eight RCTs (including 2534 participants) investigating the effects of 12 interventions for IDA treatment among school-going children in India were obtained. The results of reference-based forest plots and *P* score indicated that iron-rich fish powder was the most effective intervention for increasing Hb levels (MD: 2.07 g/dL, 95% CI: 0.68–3.47, *P* score=0.8656), followed by iron and folic acid (IFA) given twice weekly (MD: 1.47 g/dL, 95% CI: -0.31–3.25, *P* score=0.7209). Additionally, IFA supplementation twice weekly was found to be highly effective in increasing serum ferritin levels among anemic school children (MD: 0.80 ng/mL, 95% CI: 0.33–1.27, *P* score=0.9148).

**Conclusion:** It seems that iron-rich fish powder and intermittent IFA supplementation were the most effective interventions, but further research is needed to confirm these results and assess their public health implications.

**Protocol Registration:** PROSPERO registration number was CRD42024541802.

## Background

 Iron-deficiency anemia (IDA) is a significant global health issue, affecting a large portion of the population worldwide. IDA is a condition in which low levels of iron are associated with anemia and the presence of microcytic hypochromic red cells. One-fourth of the global population is estimated to be anemic, and in 2021, approximately 1.92 billion people were globally estimated to have anemia, with a substantial proportion attributed to iron deficiency.^1^ This condition is particularly prevalent among school-going children, with varying prevalence reported across different regions.

 In India, the prevalence of IDA among school children varies widely, ranging from 27% to 90%, according to different studies.^2^ The prevalence of anemia in India exceeds that of many other developing countries.^3^ According to the National Family Health Survey-5 (2019–2021), the prevalence of anemia among adolescents aged 15–19 years was 31.1% in boys and 59.1% in girls. Anemia prevalence shows pronounced state-level disparities in India and is rising across all age cohorts, including school-aged children.^4^

 This variability underscores the complex nature of the problem within the country’s diverse demographics and socioeconomic contexts. IDA not only compromises children’s physical growth and cognitive development but also adversely affects their educational outcomes and overall quality of life.^5,6^

 In India, multiple government-led interventions have been implemented to address IDA, particularly through the Anaemia Mukt Bharat strategy. They include prophylactic iron and folic acid (IFA) supplementation across different age and physiological groups, biannual deworming to reduce parasite-related anemia, and behavior change communication to improve compliance and awareness.^7-15^

 While several individual studies and traditional meta-analyses have evaluated the impact of interventions addressing IDA, a significant gap persists regarding the comprehensive comparison of their relative effectiveness. Network meta-analysis (NMA) uniquely addresses this limitation. Unlike conventional pairwise meta-analysis, which compares interventions two at a time, NMA facilitates simultaneous evaluation and ranking of multiple interventions, even in the absence of direct comparisons between each pair. This analytical approach is particularly valuable for evidence-based decision-making in settings such as India, characterized by multiple, overlapping interventions.

 This study aims to bridge this critical gap by quantifying and ranking the effectiveness of various interventions targeting IDA among school-going children in India. The outcomes will provide essential insights to inform and optimize future public health strategies and interventions.

## Methods

 The protocol of this review was duly registered by the International Prospective Register of Systematic Reviews (with registration No. CRD42024541802), and the review was conducted and reported following the systematic review reporting standards recommended by the Preferred Reporting Items for Systematic Reviews and Meta-Analyses (PRISMA)-NMA guidelines.^17^

###  Systematic search

 Searches were conducted in PubMed, Scopus, and ScienceDirect databases using several keywords, including ‘iron deficiency anemia’, ‘interventions’, ‘school-aged children’, ‘hemoglobin’, ‘ferritin’, and ‘India’, and two authors independently screened paper titles and abstracts. In addition to reading the full texts of all potentially relevant articles, the reviewers screened the reference lists of relevant meta-analyses. It should be noted that the systematic search was restricted to articles published in English.

###  Study selection and eligibility criteria

*Participants:* Participants eligible for the study included anemic school children aged between 5 and 19 years living in India. There were no specific gender restrictions.

*Study design:* Randomized controlled trials (RCTs published from May 1948 to June 2024) reporting various interventions on anemia in India were included in this meta-analysis. On the other hand, cross-over trials without a washout period, cluster RCTs, case studies, qualitative studies, and single-case series were excluded from the analysis.

*Outcome variables:* Based on a systematic search in the literature and expert consultation, hemoglobin (Hb) and serum ferritin were selected as outcome measures for the NMA. Anemia was defined as Hb < 110 g/L for children, with gender-specific cutoffs of < 130 g/L for males and < 120 g/L for females. Iron deficiency was defined as serum ferritin < 15 µg/L.

###  Screening and data extraction

 Two reviewers independently extracted trial characteristics, including the first author’s last name, year of publication, study design, number of participants, age group, gender, intervention and comparator characteristics, duration and dosage of intervention, and the mean and standard deviation (SD) of change from baseline Hb/ferritin in each study group. Disagreements were resolved by consulting the third author.

###  Risk of bias (quality) assessment

 Two authors independently assessed the risk of bias (RoB) of the RCTs as per the guidance outlined in version 2.0 of the Cochrane tool for the RoB assessment tool for randomized trials (RoB 2).^18^ The RoB for each included study was evaluated across the five standard domains of the tool, such as (1) bias arising from the randomization process, (2) bias due to deviations from intended interventions, (3) bias due to missing data, (4) bias in the measurement of the outcome, and (5) bias in the selection of the reported result.

###  Data synthesis and analysis

 Mean differences (MDs) and their 95% confidence intervals (CIs) were considered the effect size for reporting results. To perform the analyses, the means and SD of change in Hb and serum ferritin from the baseline in each study group were calculated. If these values were not reported, the Cochrane Handbook guidance was followed to determine them using baseline and endpoint measures,^19^ estimating the SD of change based on the baseline and final SDs, along with an assumed correlation coefficient of 0.5.

 Network geometry was assessed by plotting a network plot. A random-effect model for Hb and fixed-effects model for ferritin with a frequentist framework were conducted to compute the network estimates for each outcome.^20,21^ The results were presented using net league tables. *P* scores were obtained to determine the relative ranking of interventions.^22^ The *P* score ranged from 0 to 1, with higher scores indicating more effective interventions.

 The node-splitting approach was used to calculate indirect estimates and evaluate consistency/incoherence between direct and indirect estimates.^19^ In addition, transitivity was assessed by examining two key effect modifiers (i.e., age and gender) across the five trials that informed our indirect comparisons. Sensitivity analyses were performed by excluding studies with low sample sizes and studies with severe anemic levels or those that did not mention the level of anemia. A comparison-adjusted funnel plot and Egger’s test were created to assess potential publication bias.^23^ Given only eight studies, the supplement’s funnel plot offers limited insight, so any conclusions on publication bias remain tentative. All analyses were performed in R (version 4.4.1)^24^ using the Netmeta package and the NMA Studio online platform.

## Results

###  Literature search results and study selection process

**Figure 1:**
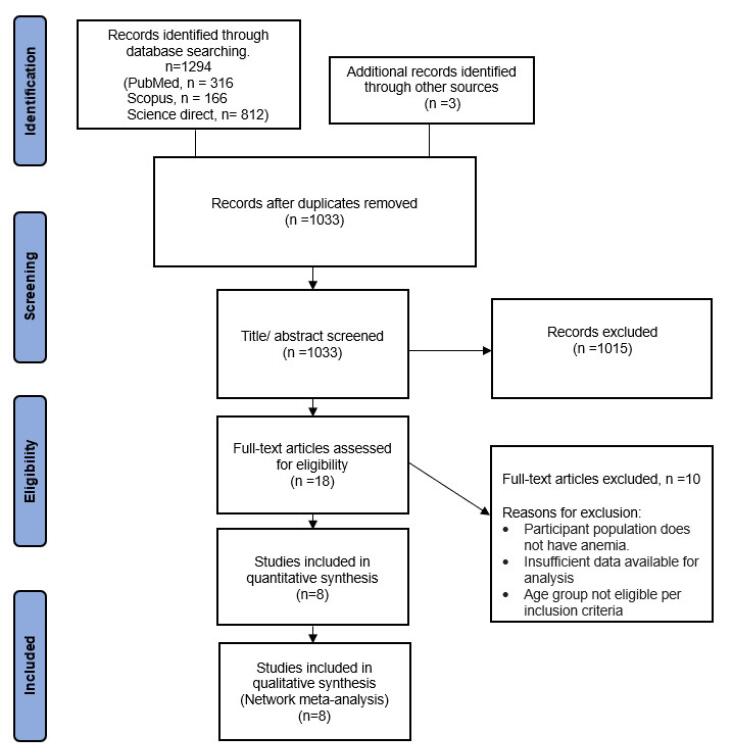
study selection process outlined using PRISMA flow diagram. *Note*. PRISMA: Preferred Reporting Items for Systematic Reviews and Meta-Analyses

 The process of selecting studies is illustrated in Figure 1 using a PRISMA flow diagram. A total of 1,297 references were identified from the literature research, and 264 duplicates were removed. After screening 1,015 out of 1,033 records were excluded as they did not meet our inclusion criteria. The full texts of the remaining 18 records were reviewed of which, 8 trials^8-15^ were considered eligible for inclusion.

###  Characteristics of the included trials

 In total, eight RCTs^8-15^ with 2534 school-going children were eligible for inclusion in the present NMA (Supplementary file 1, Table S1). The included trials were published between 1982 and 2021. The sample size of the trials ranged from 28 to 1167. The follow-up durations ranged from seven to forty weeks. Overall, 5 (62.5%), 1 (12.5%), and 2 (25%) trials were conducted on girls, boys, and either gender, respectively. The ages of the children ranged from five to nineteen years. Seven studies (87.5%) were reported from northern India, and one study (12.5%) was from southern India. Four studies (50%) were conducted on mild and moderate anemic school-going children, while one study (12.5%) included mild, moderate, and severe anemic children. Anemia levels were not specified in three studies (37.5%).

###  Risk of bias in the included trials

 The overall RoB of the included studies as assessed by the Cochrane RoB 2 tool is summarized in Table S2. Five trials (62.5%) demonstrated low RoB, and three trials (37.5%) were rated as having some concerns.

###  Network Meta-Analysis for the Outcome Hemoglobin 

**Figure 2:**
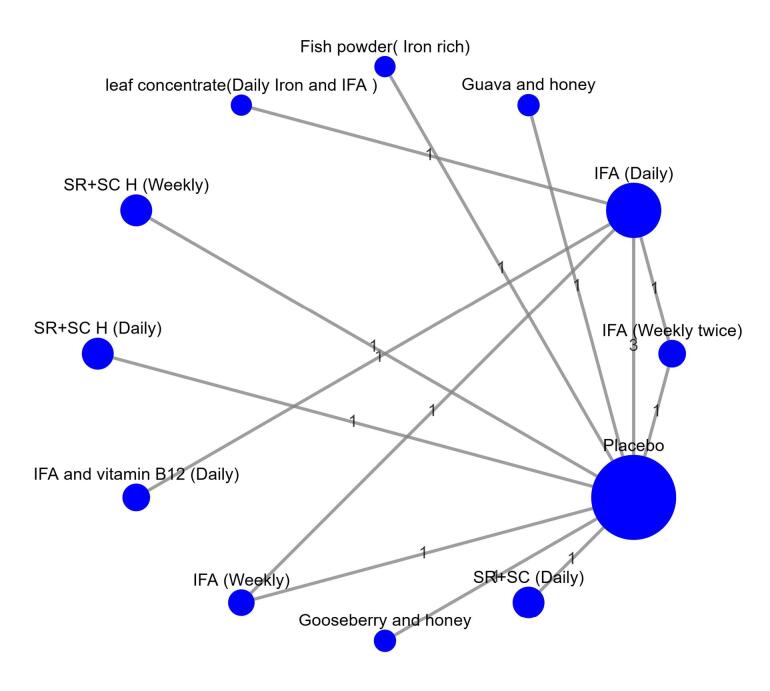
The network plot (outcome: Hb) Node sizes representing the total number of participants allocated to each intervention and line thicknesses indicating the number of trials evaluating each direct comparison. *Note*. Hb: Hemoglobin; IFA: Iron and folic acid; SR: Sootshekhar Rasa; SC: Sitopaladi Churna

 Eight studies^8-15^ were included in the NMA comparing 12 treatments (Figure 2). Node splitting analysis was conducted to assess the consistency of the network. All *P* values for comparisons between direct and indirect evidence were found to be greater than 0.05, indicating that the assumption of consistency was not violated (Figure S1 and Table S3). Our indirect comparisons were derived from five trials enrolling exclusively school-aged children (5–19 years), thereby eliminating age heterogeneity; one trial included no females, and the others were predominantly or exclusively female (100%, 100%, 100%, and 84%). There is no plausible biological basis for gender to alter Hb response in this population; therefore, it was assumed that there is no evidence of transitivity violation.

**Figure 3:**
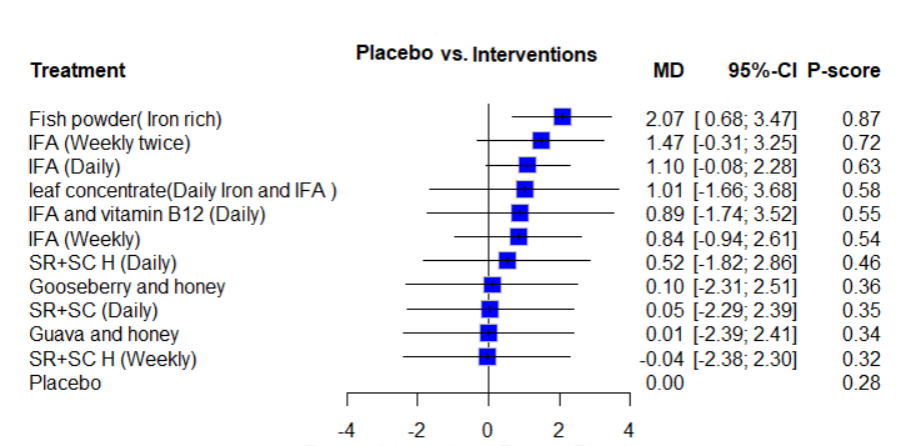
Reference-based forest plot comparing treatments with a placebo [outcome: Hb (g/dL)]. *Note*. Hb: Hemoglobin; IFA: Iron and folic acid; SR: Sootshekhar Rasa; SC: Sitopaladi Churna; MD: Mean difference; CI: Confidence interval

 A random effects model was chosen due to high heterogeneity (I^2^ statistic = 98.5%, CI: 98.0–99.0). The NMA revealed that interventions such as iron-rich fish powder and IFA (twice weekly) showed the highest MD in Hb levels compared to a placebo (Figure 3). Specifically, iron-rich fish powder and IFA (twice weekly) had an MD of 2.07 g/dL (95% CI: 0.68–3.47) and 0.80 g/dL (95% CI: 0.33–1.27) higher in Hb levels, respectively, compared to a placebo.

 Detailed direct, indirect, and network estimates for the effects of various interventions are indicated in the Net League table (Table S4).

 Based on *P* scores, iron-rich fish powder had the highest score of 0.8656, followed by IFA (weekly twice), with a score of 0.7209. The lowest *P* scores were for Sootshekhar Rasa (250 mg) and Sitopaladi Churna (400 mg) (weekly), respectively (Figure 3).

###  Sensitivity analysis 

 Sensitivity analyses were conducted by excluding studies with low sample sizes and those with severe anemia levels or the ones in which the anemia level was not specified. These analyses demonstrated that the main meta-analysis results remained robust and unaffected by these exclusions (Figures S2 and S3 and Tables S5 and S6).

###  Publication bias 

 Publication bias was assessed using a funnel plot (Figure S4) and Egger’s test. Egger’s test indicated no evidence of small-study effects (t = 0.30, *P* = 0.7650). However, given the limited number of included studies (n = 8), the funnel plot was provided as a supplementary visual aid. Accordingly, definitive conclusions regarding publication bias should be interpreted with caution.

###  Network meta-analysis for the outcome serum ferritin

**Figure 4:**
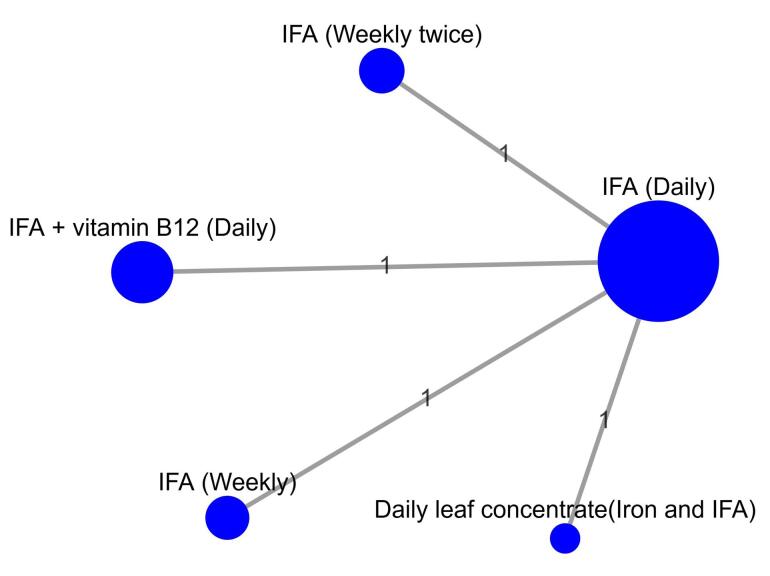
Network plot (outcome: serum ferritin). *Note*. IFA: Iron and folic acid

 For the outcome serum ferritin, three studies^10,12,13^ were included in the NMA comparing five treatments (Figure 4). A fixed-effects model was chosen due to a low heterogeneity (I^2^ statistic = 0%). Based on the NMA, IFA (twice weekly) revealed the highest MD of 0.80 ng/mL (95% CI: 0.33–1.27) in serum ferritin levels compared to IFA (daily) among anemic school-going children. Based on *P* scores, IFA (twice weekly) had the highest score of 0.9148 (Figure S5).

 Detailed direct, indirect, and network estimates for the effects of different interventions are provided in Net league table (Table S7).

## Discussion

 The present NMA synthesized evidence from eight RCTs involving a total of 2,534 participants to assess the effectiveness of various interventions for treating IDA in school-going children. The results of this analysis highlight several key findings that have important implications for public health strategies aimed at addressing IDA.

 The analysis confirmed that iron-rich fish powder and intermittent IFA supplementation (administered twice weekly) were the most effective interventions in improving Hb levels compared to a placebo (MDs of 2.04 g/dL and 1.47 g/dL, respectively). The robustness of these findings is supported by the node-splitting analysis, which indicated a strong agreement between direct and indirect evidence. Additionally, *P* scores reinforced that iron-rich fish powder (0.8042) and IFA (twice weekly) (0.7197) were the top interventions for enhancing Hb levels. According to Alleyne et al, the high-dose iron supplements are poorly absorbed, prone to oxidative damage, and often cause side effects.^25^ NaFeEDTA-enriched fish powder overcomes these issues by offering greater bioavailability even with dietary inhibitors, such as phytic acid and minimal oxidative damage or adverse effects.^26^

 In terms of serum ferritin, a marker of iron storage, the findings represented that IFA (twice weekly) was the most effective intervention, followed by IFA (weekly). The MDs in serum ferritin levels between these interventions and daily IFA supplementation were 0.80 ng/mL and 0.10 ng/mL, respectively, with *P* scores of 0.9148 for IFA (twice weekly) and 0.5045 for IFA (weekly). These results suggest that intermittent supplementation, particularly twice-weekly IFA, may be more effective than daily supplementation for improving iron stores in children.

 A major strength of this study was the rigorous methodology employed, including the independent review of studies by two reviewers and the use of the Risk of Bias tool (RoB 2) to assess the quality of the included studies. The node-splitting analysis further enhanced the credibility of the findings by ensuring concordance between direct and indirect evidence. However, the study also had limitations that should be acknowledged. Out of the eight included studies, five were assessed as having a low RoB, while three had some concerns, potentially affecting the reliability of the overall results. Moreover, the relatively small number of studies included in the NMA may have limited the generalizability of our findings, and the constructed network was unable to estimate indirect evidence among various interventions comprehensively. Furthermore, the review was restricted to English-language publications due to resource constraints, which may have led to the exclusion of potentially relevant studies and introduced selection bias.

 Nonetheless, the findings of this study have significant implications for public health interventions targeting IDA among school-going children, particularly in regions such as India, where anemia remains a major health concern. The superior efficacy of iron-rich fish powder and intermittent IFA supplementation underscores the need for policymakers to prioritize these interventions in anemia control programs. Integrating these effective strategies into existing nutritional and health programs could substantially improve the iron status and overall health of children, contributing to better educational outcomes and long-term wellbeing. Further research should investigate the acceptability, feasibility, and cost-effectiveness of these interventions across different cultural and socioeconomic contexts. This would provide valuable insights for scaling up these strategies and ensuring their sustainability in diverse settings.

**Highlights**

- This network meta-analysis (NMA) evaluated various interventions for treating iron-deficiency anemia (IDA) in school-aged children in India.
- Iron-rich fish powder and intermittent iron-folic acid supplementation emerged as the most effective strategies.
- Further randomized trials should confirm these results and shape public health policy and practice.

## Conclusion

 This NMA provides important evidence on the effectiveness of different interventions for treating IDA in school-going children. While iron-rich fish powder and intermittent IFA supplementation emerged as the most effective strategies, future research is required to confirm these findings and explore their broader implications for public health policy and practice.

## Acknowledgments

 The authors gratefully acknowledge the support, infrastructure, and facilities provided by ICMR-NITM, Belagavi, and MRHRU, Sirwar.

## Competing Interests

 The authors declare that they have no competing interests.

## Ethical Approval

 Not applicable.

## Funding

 Not applicable.

## Supplementary Files

Supplementary file 1 contains Tables S1-S7 and Figures S1-S5.
