# Supplementary material for: Effect of Interventions on Iron-Deficiency Anemia Among School-Going Children in India: A Systematic Review and Network Meta-Analysis
Source: J Res Health Sci. 2025 Jun 10;25(3):e00651. doi: 10.34172/jrhs.8985 (PMC12445883; doi:10.34172/jrhs.8985)
Supplement: Supplementary file 1 — contains Tables S1-S7 and Figures S1-S5. [file jrhs-25-e00651-s001.pdf]

JRHS
Journal of Research in Health Sciences
doi:10.34172/jrhs.8985
JRHS 2025; 25(3):e00651

Supplementary file 1

Table S1: Characteristics of Included Studies

No.	Author	Study design	No of Arms	Arms with dose	Level of Anemia	Mean Difference	Standard deviation	Number of subjects	Age group	Female Percentage	Year Of publication
1	[8] Kunnath et al., 2021	Randomised double-blinded, controlled intervention trial	2	Fish powder (Iron rich) (250 mg/100 g)	Mild	2.5	0.96	33	10 to 19	100	2021
				Placebo		0.1	0.4	33			
			2	Fish powder (Iron rich) (250 mg/100 g)	Moderate	2.2	1.42	26	10 to 19	100	
				Placebo-moderate		-0.2	1.05	26			
			2	Fish powder (Iron rich) (250 mg/100 g)	Severe	1.5	0.43	3	10 to 19	100	
				Placebo		0.1	0.3	2			
2	[9] Sen et al., 2012	Experimental-control semi longitudinal study	4	IFA (Daily) (100mg Fe, 0.5mg folic acid)	Not mentioned	0.92	1.07	34	9 to 13	100	2012
				IFA (weekly twice) (100mg Fe, 0.5mg folic acid)		0.95	1.03	61			
				IFA (Weekly) (100mg Fe, 0.5mg folic acid)		0.61	0.65	46			
				Placebo		0.50	0.22	30			
3	[10] Bansal et al., 2015	Community-based randomized controlled	2	IFA (Daily) (100 mg Fe, 500µg folic acid)	Mild and moderate	0.97	1.37	184	11 to 18	100	2015
				IFA + vitamin B12 (Daily)		0.76	1.14	189			

		trial		(100mg Fe, 500µg folic acid and cyanocobalamin)							
4	[11] Seshadri et al., 1982	Randomized controlled trial	2	IFA (Daily)<br>(40mg Fe, 0.2 mg folic acid)	Not mentioned	2.42	0.29	14	5 to 6	0	1982
				Placebo		-0.09	0.33	14			
5	[12] Gupta et al., 2014	Randomized controlled trial	3	IFA (Daily)<br>(100 mg Fe, 500 µg of folic acid)	Mild and moderate	2.30	1.4	111	11 to 19	100	2014
				IFA (weekly)<br>(100 mg Fe and 500 µg folic acid)		2.40	1.90	108			
				IFA (weekly twice)<br>(100 mg Fe and 500 µg folic acid)		3.10	2.20	112			
6	[13] Vyas et al., 2006	Randomized controlled trial	2	IFA(Daily)<br>(60mg Fe, 500µg folic acid)	Mild, moderate and severe	1.54	1.21	40	14 to 18	100	2006
				leaf concentrate (Daily)<br>(5mg Fe, 13µg folic acid)		1.45	1.23	46			
7	[14] Prakash et al., 2010	Single-blinded, randomized, controlled study.	5	Sootshekhar Rasa (SR)(125mg) and<br>Sitopaladi Churna (SC) (500mg) (Daily)	Mild and moderate	0.23	0.45	245	11 to 18	84.4	2010
				Sootshekhar Rasa (SR)(250mg) and Sitopaladi Churna (SC) (400mg)<br>(Daily)		0.70	0.61	233			
				Sootshekhar Rasa (SR) (250mg) and Sitopaladi Churna (SC) (400mg)<br>(Weekly)		0.14	0.50	220			
				IFA (Daily)<br>(100mg Fe, 500µg folic acid)		0.37	0.56	215			
				Placebo		0.18	0.43	254			
8	[15] Reeta et al., 2018	Randomized controlled trial	3	Gooseberry juice and honey (30ml)	Not mentioned	0.11	2.04	85	12 to 16	100	2018
				Guava juice and honey		0.02	1.83	85			

				(30 ml)							
				Placebo		0.01	1.83	85			

Table S2: Details of Risk of Bias

Study ID	Randomization	Deviations from intended interventions	Missing data	Outcome measurement	Selection of reported result	Overall
[8] Kunnath et al., 2021	Low	Low	Low	Low	Some concerns	Low
[9] Sen et al., 2012	Some concerns	Low	Low	Low	Some concerns	Low
[10] Bansal et al., 2015	Low	Low	Low	Low	Some concerns	Low
[11] Seshadri et al.,1982	Some concerns	Some concerns	Low	Low	Some concerns	Some concerns
[12] Gupta et al., 2014	Some concerns	Some concerns	Some concerns	Low	Some concerns	Some concerns
[13] Vyas et al., 2006	Some concerns	Low	High	Low	Some concerns	Some concerns
[14] Prakash et al.,2010	Low	Some concerns	Low	Low	Some concerns	Low
[15] Reeta et al., 2018	Low	Low	Low	Low	Some concerns	Low

Table S3: Result of Node Splitting Analysis

Comparison	k	prop	nma	direct	indir.	Diff	z	P-value
IFA (Daily): IFA (Weekly twice)	1	0.56	-0.37	-0.80	0.17	-0.97	-0.53	0.5965
IFA (Daily): IFA (Weekly)	1	0.56	0.27	-0.10	0.72	-0.82	-0.45	0.6518
IFA (Daily): Placebo	3	0.75	1.10	1.36	0.32	1.04	0.75	0.4545
IFA (Weekly twice): Placebo	1	0.57	1.47	1.05	2.02	-0.97	-0.53	0.5965
IFA (Weekly): Placebo	1	0.57	0.84	0.48	1.30	-0.82	-0.45	0.6518

k-Number of comparisons
prop- proportion of direct evidence
nma- effect estimate by mixed comparison
direct-effect estimate by direct comparison

Table S4: Net league Table for Outcome haemoglobin

Fish powder (Iron rich)	1.97 (-0.77, 4.72)	2.06 (-0.68, 4.8)	1.18 (-1.75, 4.11)	0.97 (-0.83, 2.77)	1.24 (-0.98, 3.45)
-1.97 (-4.71, 0.77)	Gooseberry and honey	0.09 (-3.27, 3.45)	-0.79 (-4.31, 2.73)	-1.00 (-3.65, 1.64)	-0.74 (-3.68, 2.21)
-2.06 (-4.79, 0.67)	-0.09 (-3.45, 3.27)	Guava and honey	-0.88 (-4.39, 2.63)	-1.09 (-3.73, 1.55)	-0.83 (-3.77, 2.12)
-1.18 (-4.11, 1.75)	0.79 (-2.73, 4.31)	0.88 (-2.63, 4.39)	IFA and vitamin B12 (Daily)	-0.21 (-2.53, 2.11)	0.06 (-2.85, 96)
-0.97 (-2.76, 0.83)	1.00 (-1.64, 3.65)	1.09 (-1.55, 3.73)	0.21 (-2.11, 2.53)	IFA (Daily)	0.27 (-1.48, 2.01)
-1.24 (-3.45, 0.98)	0.74 (-2.22, 3.69)	0.83 (-2.12, 3.77)	-0.06 (-2.96, 2.85)	-0.27 (-2.01, 1.48)	IFA (Weekly)
-0.60 (-2.82, 1.62)	1.37 (-1.58, 4.32)	1.46 (-1.49, 4.40)	0.58 (-2.33, 3.48)	0.37 (-1.39, 2.12)	0.63 (-1.70, 2.7)
-1.06 (-4.03, 1.91)	0.91 (-2.63, 4.46)	1.00 (-2.54, 4.54)	0.12 (-3.19, 3.43)	-0.09 (-2.45, 2.27)	0.18 (-2.76, 3.11)
-2.07 (-3.44, -0.70)	-0.10 (-2.48, 2.28)	-0.01 (-2.38, 2.36)	-0.89 (-3.48, 1.70)	-1.10 (-2.26, 0.06)	-0.84 (-2.58, 0.91)
-2.02 (-4.70, 0.66)	-0.05 (-3.36, 3.26)	0.04 (-3.26, 3.34)	-0.84 (-4.31, 2.63)	-1.05 (-3.63, 1.53)	-0.79 (-3.67, 2.10)
-1.55 (-4.23, 1.12)	0.42 (-2.89, 3.73)	0.51 (-2.79, 3.81)	-0.37 (-3.84, 3.08)	-0.58 (-3.16, 2.00)	-0.32 (-3.20, 2.57)
-2.11 (-4.79, 0.57)	-0.14 (-3.45, 3.17)	-0.05 (-3.35, 3.25)	-0.93 (-4.40, 2.54)	-1.14 (-3.72, 1.44)	-0.88 (-3.76, 2.01)

0.60 (-1.62, 2.82)	1.06 (-1.91, 4.03)	2.07 (0.70, 3.44)	2.02 (-0.66, 4.70)	1.55 (-1.13, 4.23)	2.11 (-0.57, 4.79)
-1.37 (-4.32, 1.58)	-0.91 (-4.46, 2.63)	0.10 (-2.28, 2.48)	0.05 (-3.26, 3.36)	-0.42 (-3.73, 2.89)	0.14 (-3.17, 3.45)
-1.46 (-4.40, 1.49)	-1.00 (-4.54, 2.54)	0.01 (-2.36, 2.38)	-0.04 (-3.34, 3.26)	-0.51 (-3.81, 2.79)	0.05 (-3.25, 3.35)
-0.58 (-3.49, 2.33)	-0.12 (-3.43, 3.19)	0.89 (-1.70, 3.48)	0.84 (-2.63, 4.31)	0.37 (-3.10, 3.84)	0.93 (-2.54, 4.40)
-0.37 (-2.12, 1.39)	0.09 (-2.27, 2.45)	1.10 (-0.06, 2.26)	1.05 (-1.53, 3.63)	0.58 (-2.00, 3.16)	1.14 (-1.44, 3.72)
-0.63 (-2.97, 1.70)	-0.18 (-3.11, 2.76)	0.84 (-0.91, 2.58)	0.79 (-2.10, 3.67)	0.32 (-2.57, 3.20)	0.88 (-2.01, 3.76)
IFA (Weekly twice)	0.46 (-2.48, 3.40)	1.47 (-0.278, 3.22)	1.42 (-1.47, 4.31)	0.95 (-1.94, 3.84)	1.51 (-1.38, 4.40)
-0.46 (-3.40, 2.48)	leaf concentrate (Daily Iron and IFA)	1.01 (-1.62, 3.64)	0.96 (-2.54, 4.46)	0.49 (-3.01, 3.99)	1.05 (-2.45, 4.55)
-1.47 (-3.22, 0.28)	-1.01 (-3.64, 1.62)	Placebo	-0.05 (-2.35, 2.25)	-0.52 (-2.82, 1.78)	0.04 (-2.26, 2.34)
-1.42 (-4.31, 1.47)	-0.96 (-4.46, 2.54)	0.05 (-2.25, 2.35)	SR+SC (Daily)	-0.47 (-3.73, 2.79)	0.09 (-3.17, 3.35)
-0.95 (-3.84, 1.94)	-0.49 (-3.99, 3.01)	0.52 (-1.78, 2.82)	0.47 (-2.79, 3.73)	SR+SC H (Daily)	0.56 (-2.70, 3.82)
-1.51 (-4.40, 1.38)	-1.05 (-4.55, 2.45)	-0.04 (-2.34, 2.26)	-0.09 (-3.35, 3.17)	-0.56 (-3.82, 2.70)	SR+SC H (Weekly)

Table S5: Sensitivity Analysis, P-Scores Based on Sample Size

Interventions	P-Score
Fish powder (Iron rich)	0.9924
IFA (Weekly twice)	0.8430
IFA (Daily)	0.6042
IFA (Weekly)	0.5813
SR+SC H (Daily)	0.5392
leaf concentrate (Daily) (Iron and IFA)	0.5276
IFA and vitamin B12 (Daily)	0.4707
Gooseberry and honey	0.3370
SR+SC (Daily)	0.3058
Guava and honey	0.2956
SR+SC H (Weekly)	0.2639
Placebo	0.2393

Table S6: Sensitivity Analysis, P-Scores Based on Anemia Levels

Interventions	P-Score
Fish powder (Iron rich)	1.0000
IFA (Weekly twice)	0.8686
SR+SC H (Daily)	0.7339
IFA (Weekly)	0.5497
IFA (Daily)	0.5349
SR+SC (Daily)	0.3566
Placebo	0.2081
IFA and vitamin B12 (Daily)	0.1837
SR+SC H (Weekly)	0.0644

Table S7: Net league Table for Outcome Serum Ferritin

Daily leaf concentrate (Iron and IFA)	-0.28 (-0.76, 0.20)	-4.98 (-8.98, -0.98)	-0.48 (-1.21, 0.25)	0.52 (-0.15, 1.19)
0.28 (-0.20, 0.76)	IFA (Daily)	-4.70 (-8.67, -0.73)	-0.20 (-0.75, 0.35)	0.80 (0.33, 1.27)
4.98 (0.98, 8.98)	4.70 (0.73, 8.67)	IFA + vitamin B12 (Daily)	4.50 (0.50, 8.51)	5.50 (1.51, 9.50)
0.48 (-0.25, 1.21)	0.20 (-0.35, 0.75)	-4.50 (-8.51, -0.50)	IFA (Weekly)	1.00 (0.28, 1.72)
-0.52 (-1.19, 0.15)	-0.80 (-1.27, -0.33)	-5.50 (-9.50, -1.51)	-1.00 (-1.72, -0.28)	IFA (Weekly twice)

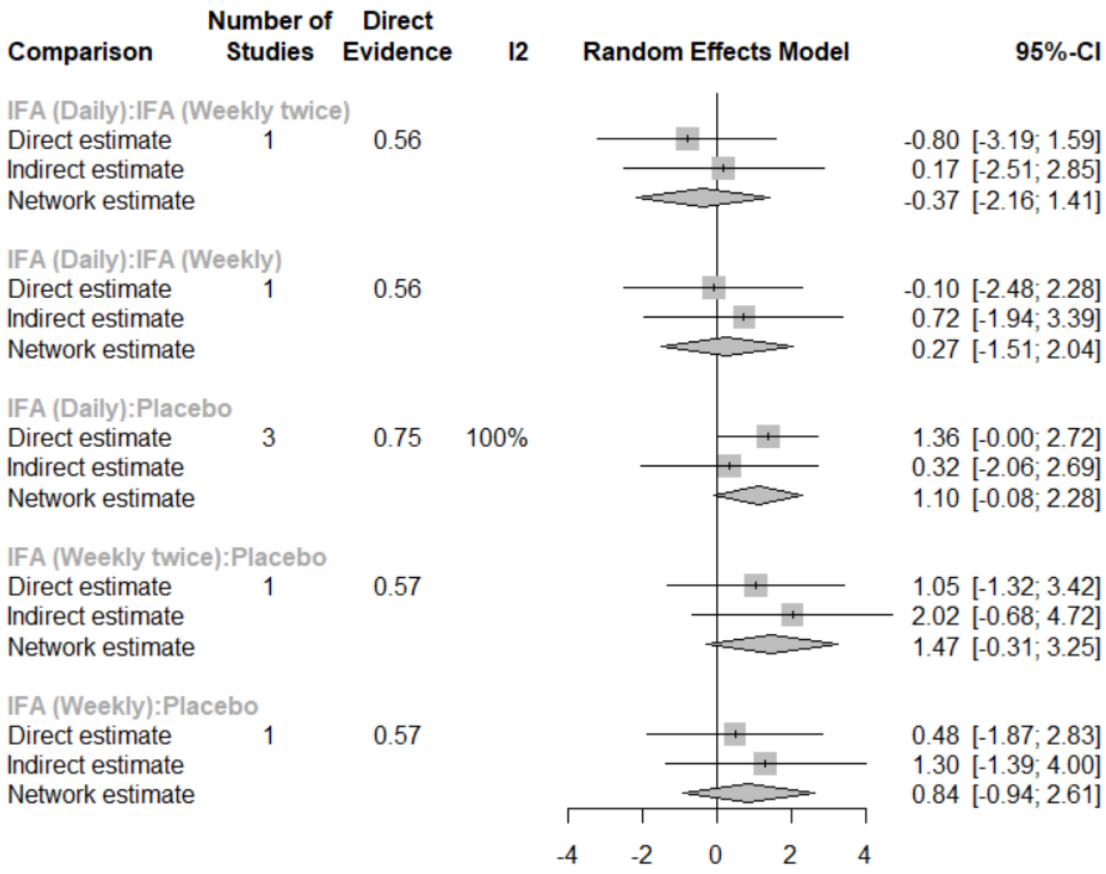

Figure S1: Result of Node Splitting Analysis

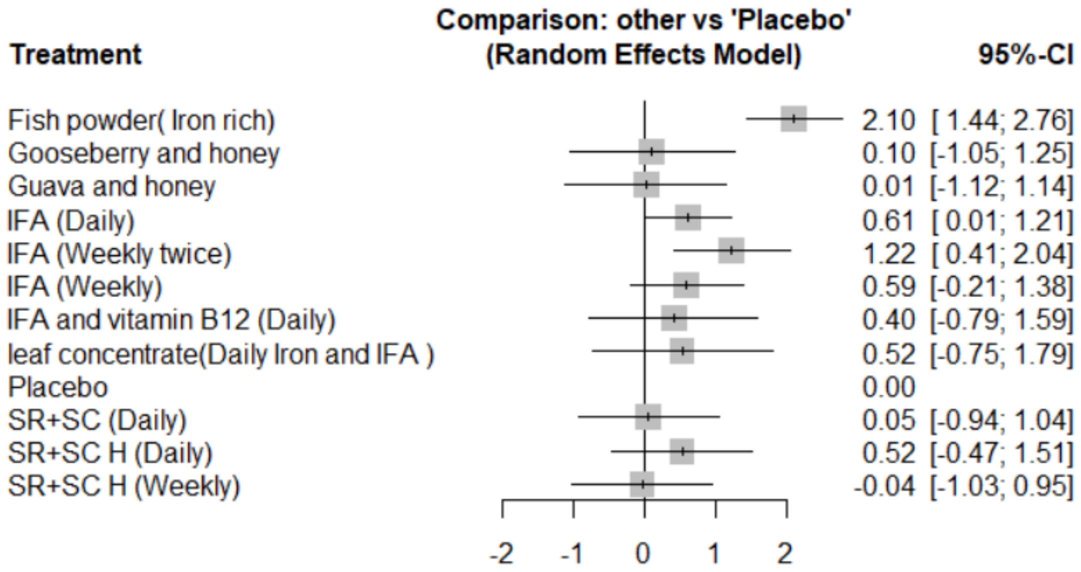

Figure S2: Forest Plot of Sensitivity Analysis Based on Sample Size

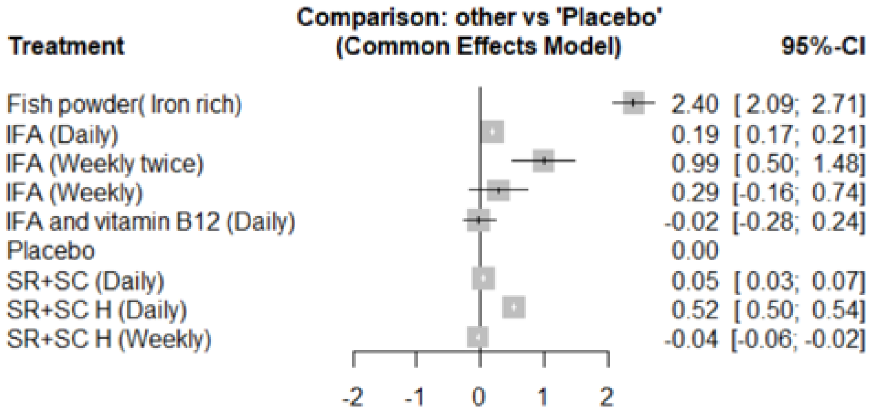

Figure S3: Forest plot of Sensitivity Analysis Based on Sample Size

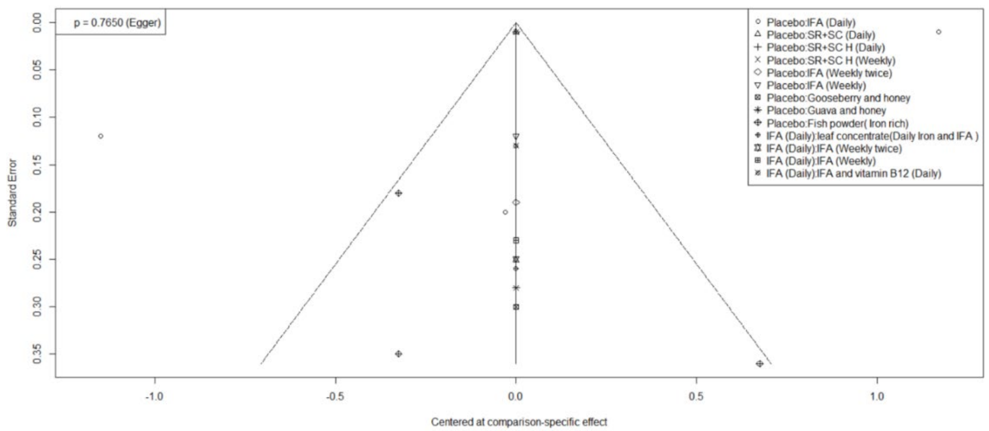

Figure S4: Funnel Plot

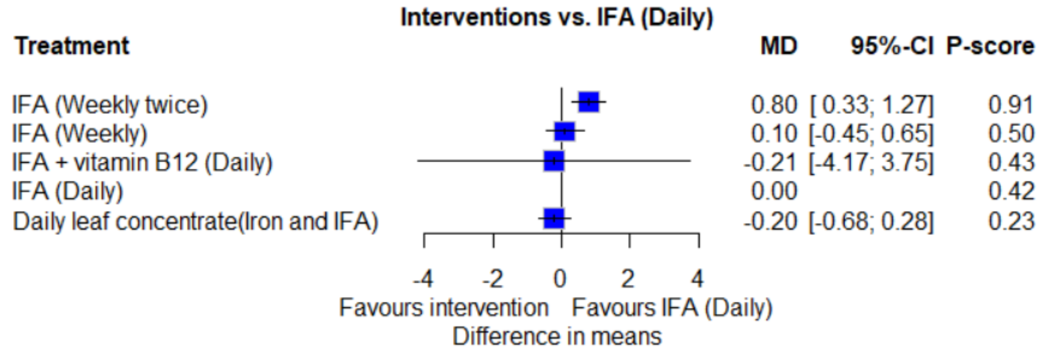

Figure S5: Reference based forest plot comparing treatments to IFA (daily) [Outcome: Serum ferritin (ng/ml)]
